# Development of species diagnostic SNP markers for quality control genotyping in four rice (*Oryza* L.) species

**DOI:** 10.1007/s11032-018-0885-z

**Published:** 2018-10-24

**Authors:** Marie Noelle Ndjiondjop, Kassa Semagn, Jianwei Zhang, Arnaud Comlan Gouda, Sèdjro Bienvenu Kpeki, Alphonse Goungoulou, Peterson Wambugu, Khady Nani Dramé, Isaac Kofi Bimpong, Dule Zhao

**Affiliations:** 1 M’bé Research Station, Africa Rice Center (AfricaRice), 01 B.P. 2551, Bouaké, 01 Côte d’Ivoire; 2 Department of Agricultural, Food and Nutritional Science, University of Alberta, 4-10 Agriculture/Forestry Centre, Edmonton, Alberta T6G 2P5 Canada; 3 Arizona Genomics Institute and The School of Plant Sciences, University of Arizona, Thomas W. Keating Bioresearch Bldg., 1657 E. Helen Street, Tucson, AZ 85721 USA; 4 Kenya Agricultural and Livestock Research Organization (KALRO), Genetic Resources Research Institute, Nairobi, Kenya; 5 AfricaRice Headquarters, 01 BP 4029, Abidjan, 01 Côte d’Ivoire

**Keywords:** African rice, Asian rice, Cultivated rice, DArTseq, Genotyping by sequencing, Wild rice

## Abstract

Species misclassification (misidentification) and handling errors have been frequently reported in various plant species conserved at diverse gene banks, which could restrict use of germplasm for correct purpose. The objectives of the present study were to (i) determine the extent of genotyping error (reproducibility) on DArTseq-based single-nucleotide polymorphisms (SNPs); (ii) determine the proportion of misclassified accessions across 3134 samples representing three African rice species complex (*Oryza glaberrima*, *O. barthii*, and *O. longistaminata*) and an Asian rice (*O. sativa*), which are conserved at the AfricaRice gene bank; and (iii) develop species- and sub-species (ecotype)-specific diagnostic SNP markers for rapid and low-cost quality control (QC) analysis. Genotyping error estimated from 15 accessions, each replicated from 2 to 16 times, varied from 0.2 to 3.1%, with an overall average of 0.8%. Using a total of 3134 accessions genotyped with 31,739 SNPs, the proportion of misclassified samples was 3.1% (97 of the 3134 accessions). Excluding the 97 misclassified accessions, we identified a total of 332 diagnostic SNPs that clearly discriminated the three indigenous African species complex from Asian rice (156 SNPs), *O. longistaminata* accessions from both *O. barthii* and *O. glaberrima* (131 SNPs), and *O. sativa* spp. *indica* from *O. sativa* spp. *japonica* (45 SNPs). Using chromosomal position, minor allele frequency, and polymorphic information content as selection criteria, we recommended a subset of 24 to 36 of the 332 diagnostic SNPs for routine QC genotyping, which would be highly useful in determining the genetic identity of each species and correct human errors during routine gene bank operations.

**Electronic supplementary material:**

The online version of this article (10.1007/s11032-018-0885-z) contains supplementary material, which is available to authorized users.

## Introduction

The Consultative Group on International Agricultural Research (CGIAR) gene banks safeguard some of the most widely used collections of crops and trees in the world, which is critical for attaining global development goals to end hunger and improve food and nutrition security. The CGIAR centers have a gene bank platform that enables them to conserve and make available germplasm under the International Plant Treaty and distribute to the global community (http://www.cgiar.org/about-us/our-programs/cgiar-genebank-platform-2017-2022). Between 2012 and 2016, CGIAR gene banks distributed over half a million accessions for research and breeding purposes across the world. The Africa Rice Center (AfricaRice) and the International Rice Research Institute (IRRI) are the CGIAR centers, which conserve rice (*Oryza* L.) germplasm. Africa harbors a huge diversity of both cultivated and wild rice species, representing six of the ten known genome types (Wambugu et al. 2013). AfricaRice conserves nearly 22,000 registered rice samples at its gene bank. The collections represent five African indigenous wild species (*Oryza barthii*, *O. longistaminata*, *O. eichingeri*, *O. punctata*, and *O. brachyantha*) and two cultivated species (*O. glaberrima* and *O. sativa*) (Ndjiondjop et al. 2017). The cultivated species of *O. glaberrima* along with the wild perennial *O. longistaminata* and wild annual *O. barthii* may be considered as a species complex (Ogbe and Williams 1978).

*O. glaberrima* accounts for approximately 14% of the collections at AfricaRice, while all other indigenous wild species are represented by smaller number of samples ranging from 1 to 125 accessions. Recently, our group used the Diversity Arrays Technology-based genotyping by sequencing (DArTseq) platform (Sansaloni et al. 2011) to characterize *O. glaberrima* collections. We observed that 44 of 2223 *O. glaberrima* accessions had unusual SNP calls and were considered outliers. Exclusion of the 44 outliers from the dataset resulted in a large decrease (by 77%) in the number of polymorphic SNPs from 16,532 in the 2223 accessions to 3834 in the remaining 2179 accessions. The 44 outlier accessions may possess rare alleles, which might make them very different from most of the *O. glaberrima* accessions. Outlier accessions might also have resulted from natural allele introgressions into *O. glaberrima* from another *Oryza* species (Jones et al. 1997; Semon et al. 2005; Orjuela et al. 2014), especially *O. barthii*, which is presumed to be the wild ancestor of the cultivated *O. glaberrima* (Ogbe and Williams 1978). In such cases, accessions identified as *O. glaberrima* might be genetically intermediate between *O. glaberrima* and *O. barthii* or other species. Human errors might also occur during (i) plant identification while collecting the accessions in their natural habitats due to inadequate taxonomic expertise and (ii) routine gene bank operations, including germplasm acquisition, conservation, regeneration/multiplication, DNA preparation, and/or genotyping. Proper taxonomic classification and identification of germplasm prior to seed banking is critically important but remains a great challenge for gene bank managers due to heavy dependence on morphological characters that are less accurate in cases where there is limited phenotypic diversity, trait ambiguity, and their variability due to genotype-by-environment interactions (Ge et al. 2001). Certain *Oryza* species are closely related, increasing the probability of misidentification, which can easily be avoided using genomic tools for accurate species identification.

Misclassification (misidentification) has been reported in several species, including *Oryza glaberrima*, *O. sativa*, and *O. barthii* (Orjuela et al. 2014), other wild rice species (Buso et al. 2001), *Dioscorea* spp. (Girma et al. 2012), and *Brassica* spp. (Mason et al. 2015). Such types of errors restrict effective use of germplasm for correct purpose in various ways. The latter includes difficulty to tell whether the “true-to-type” accession/variety has been used for line development, development of mapping populations, molecular breeding, and other genetic studies (Semagn et al. 2012). Quality control (QC) genotyping methods using low-cost, high-throughput, and user-friendly molecular markers have been developed and implemented in some species for genetic purity and genetic identity/authentication (Semagn et al. 2012; Cullingham et al. 2013; Frey et al. 2013; Curk et al. 2015; Ertiro et al. 2015; Chen et al. 2016). Species discriminating markers have also been reported in few plant species (Balasaravanan et al. 2006; Cullingham et al. 2013; Curk et al. 2015) and are finding great application in gene banks where numerous cases of misidentification have been reported (Mason et al. 2015). In rice, Kshirsagar et al. (2014) recommended 12 inter-simple sequence repeats (ISSRs) to serve as varietal diagnostic markers to assess the genetic variability of 48 *O. sativa* genotypes. Joshi et al. (2000) screened 30 ISSR markers for their polymorphism on 42 genotypes representing 17 wild *Oryza* species, *O. glaberrima* and *O. sativa*, and reported few species-specific ISSRs. Chen et al. (2017) genotyped a total of 53 samples, including *O. glaberrima* (18) and *O. sativa* (23), with 33 simple sequence repeat (SSR) markers and reported 10 SSRs that displayed different allelic profiles between the two species. Zhao et al. (2009) genotyped 103 *O. rufipogon* accessions, 10 *O. sativa* spp. *indica*, and 10 *O. sativa* spp. *japonica* cultivars with 123 intron length polymorphism (ILP) markers of which 57 of the markers were found to be highly subspecies-specific between *O. sativa* spp. *indica* and *O. sativa* spp. *japonica*. Chin et al. (2007) screened a total of 765 sequence tag sites (STS) using genomic DNA of 15 *O. sativa* spp. *indica* and 15 *O. sativa* spp. *japonica* varieties and identified 67 markers for their subspecies specificity. However, species- and subspecies-specific markers reported in previous studies are of limited value to serve as diagnostic markers for several reasons: (i) they were recommended based on very small sample size, (ii) all authors used agarose gels for fragment separation that not only has poor resolution in discriminating genotypes that differ by small allele sizes but also the method is tedious and very low throughput; and (iii) some of the markers are dominant and do not discriminate heterozygous and homozygous loci.

The availability of next-generation sequencing-based genotyping technologies, such as genotyping by sequencing (GBS) (Elshire et al. 2011) and the diversity arrays technology-based sequencing (DArTseq) platform (Sansaloni et al. 2011), have made single-nucleotide polymorphisms (SNPs) very popular for various applications. Some of the advantages of SNPs include low assay cost, high genomic abundance, bi-allelic inheritance, potential for high-throughput analysis, and relatively low genotyping error rates (Rafalski 2002; Schlotterer 2004; McCouch et al. 2012). IRRI recommended a panel of ten SNPs for low-cost QC genotyping for parent-offspring (hybridity) testing and line verification in *O. sativa* spp. *indica* genotypes (http://gsl.irri.org/genotyping/quality-control-panel/indica-rice-qc-10-snp-panel). It also suggested a panel of 24 SNPs for global QC genotyping in rice. However, the detailed methodology used in selecting the two SNP panels for QC genotyping and their relevance to serve as species and subspecies (ecotype) discriminatory marker set are not currently available. Therefore, the objectives of the present study were to (a) determine the extent of genotyping error (reproducibility) in DArTseq and the proportion of misclassified accessions across four rice species (*O. glaberrima*, *O. sativa*, *O. barthii*, and *O. longistaminata*) and (b) develop a set of species- and sub-species (ecotype)-specific diagnostic SNP markers for low-cost QC genotyping to minimize errors during routine gene bank operations.

## Materials and methods

The two initial sets of germplasm used in the present study are summarized in Supplementary Table S1. The first set consisted of 117 template control DNA samples from 15 accessions, with each accession represented between two to 16 DNA samples. The second set consisted of 3134 samples from 3097 accessions, which represent *O. longistaminata* (20), *O. barthii* (51), *O. glaberrima* (2422), and *O. sativa* (558) and genotypes derived from interspecific crosses between *O. glaberrima* and *O. sativa* (83). *O. longistaminata* was represented by smaller sample size due to limitation on the number of available collections at the AfricaRice gene bank. Thirty-seven accessions were used twice from original and regenerated seed lots to assess the level of human errors during routine gene bank operations. The interspecific genotypes, commonly referred as New Rice for Africa (NERICA), genetically resemble their recurrent *O. sativa* parents. Genomic DNA was extracted from a single plant per sample from 3-week-old seedlings grown in a screenhouse. The detailed methodology used for DNA extraction, SNP genotyping using DArTseq™, and imputation were described previously (Ndjiondjop et al. 2017). The 117 template DNA samples were randomly placed across 35 of 96-well plates used for genotyping the entire germplasm in this study. For each accession, we received 31,739 imputed SNPs from DArT Pty Ltd., Australia (http://www.diversityarrays.com), of which 82.3% of the markers (26,133 SNPs) were mapped to the 12 rice chromosomes, while the remaining 17.7% were not assigned into any of the chromosomes.

All statistical analyses were performed after filtering the SNP data of the two sets of germplasm using a minor allele frequency (MAF) of 0.01 in TASSEL v.5.2.43 software (Bradbury et al. 2007). An identity-by-state (IBS)-based genetic distance matrices were computed between pair of DNA samples of each data set using TASSEL v.5.2.43. The genetic distance matrix in the first data set was used as an indicator of genotyping error (reproducibility), whereby pairs of DNA samples from the same accession are expected to have a zero distance (no mismatch); values exceeding zero are indicative of genotyping errors with larger values showing higher proportion of mismatch between template DNA samples of the same accession. In the second dataset, the genetic distance matrix was used for cluster analysis to understand the extent of species misclassification due to human error during plant identification while collecting the accessions in the field, germplasm acquisition, and/or routine gene bank activities. Cluster analysis was performed using the neighbor-joining method implemented in molecular evolutionary genetics analysis (MEGA) v.7.0 (Kumar et al. 2016). We also used principal component analysis (PCA) implemented in TASSEL v.5.2.43. The first two principal components from the PCA were plotted for visual examination in XLSTAT 2012 (Addinsof, New York, USA; www.xlstat.com) using the scatter plot option and species/ecotype as a categorical variable. Accessions belonging to the same species/ecotype tend to cluster more closely together than those from other species/ecotype irrespective of the algorithms used for computing genetic distance matrices and the multivariate method used for analyzing genetic relationships.

For low-cost and routine quality control genotyping, diagnostic SNPs were identified from a third dataset created after excluding all misclassified accessions in the second dataset by comparing SNPs that had the same allele across all accessions of the same species/ecotype/ecology against all accessions from a second species/ecotype/ecology using an algorithm developed by the Arizona Genomics Institute and The School of Plant Sciences, University of Arizona. The selected diagnostic SNPs were then compiled into a fourth dataset for rapid navigation and comparisons between species/ecotype/ecologies using TASSEL v.5.2.43 and Flapjack v1.16.10.31 (Milne et al. 2010).

## Results

### Genotyping error and accession misclassification

**Fig. 1:**
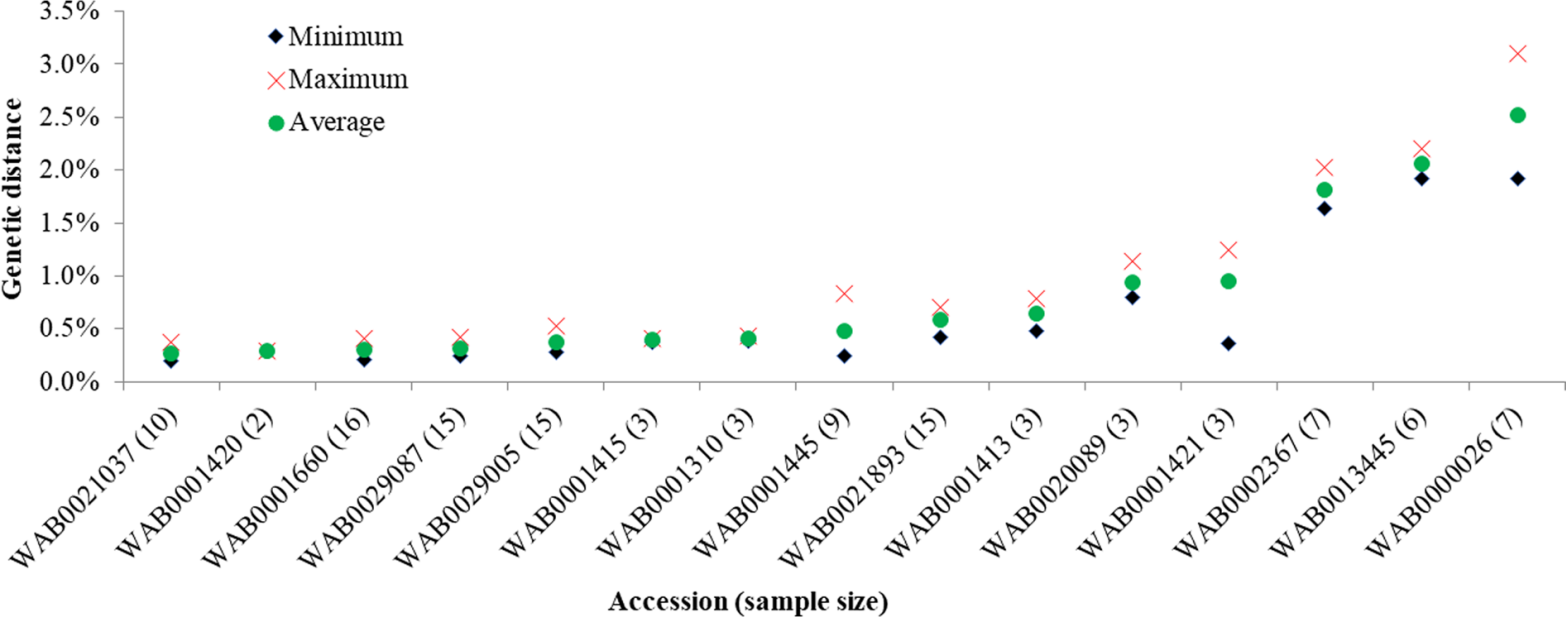
Comparison of identity-by-state-based genetic distance among pairs of DNA samples from the same accession as a measure of genotyping error of 117 DNA samples from 15 accessions. Each accession was represented from 2 to 16 DNA samples and genotyped with 31,7369 SNPs of which 23,490 SNPs were polymorphic. Only three accessions showed genotyping error between pairs of DNA samples greater than 1%

**Fig. 2:**
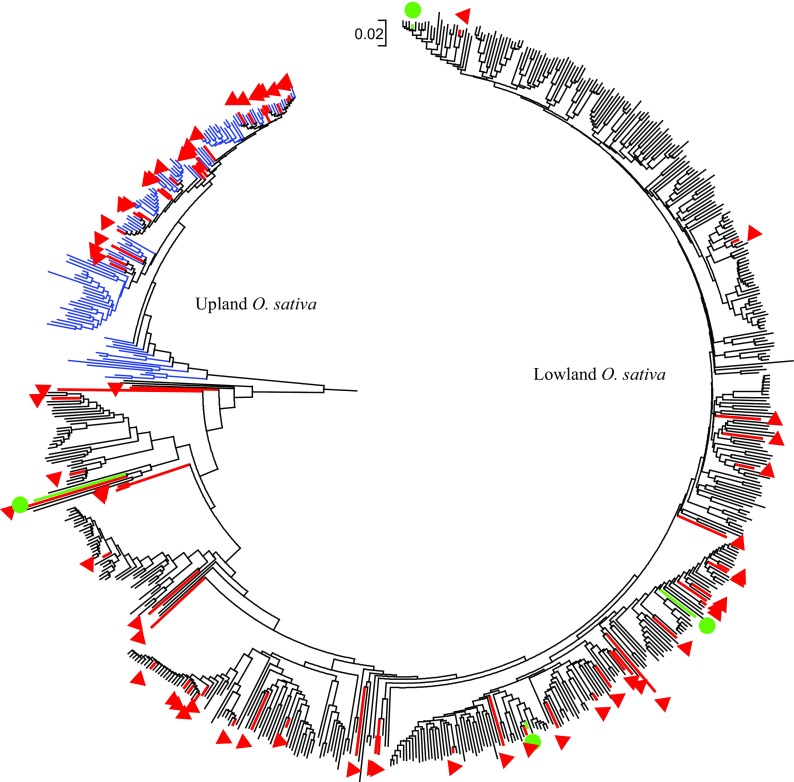
Neighbor-joining tree of 619 lowland (black) and upland (blue) *O. sativa* accessions based on genetic distance matrix computed from 27,645 polymorphic SNPs to demonstrate 64 misclassified *O. glaberrima* (red) and 4 *O. barthii* (green) accessions. Thirty-two of the 64 misclassified *O. glaberrima* accessions originated from regenerated seed lots

**Fig. 3:**
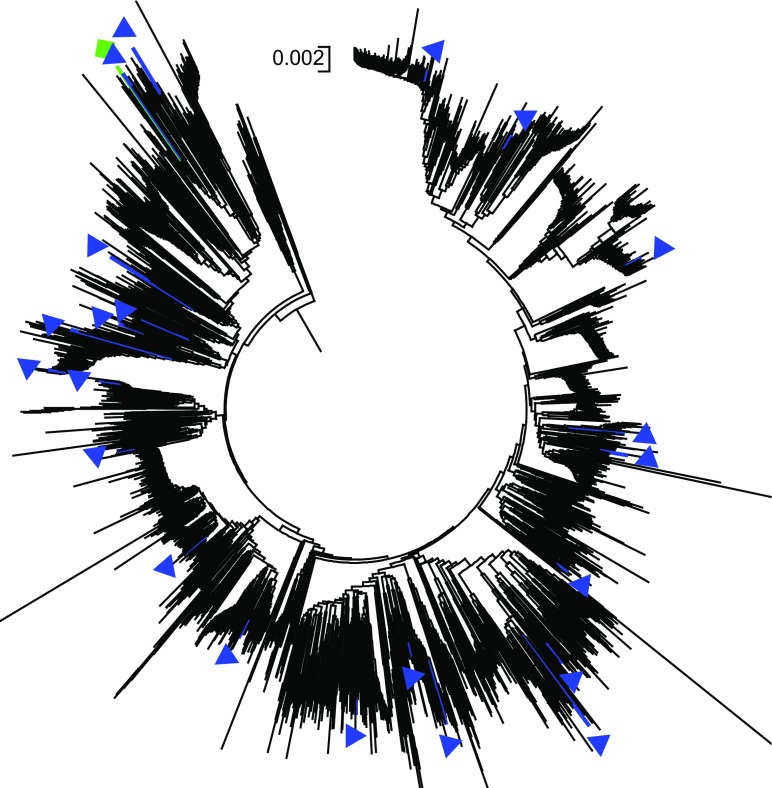
Neighbor-joining tree of *O. glaberrima* (black) accessions with misclassified *O. sativa* (blue) and *O. barthii* (green) accessions based on genetic distance matrix computed from 27,645 polymorphic SNPs

After filtering the SNPs using a minor allele frequency of 1%, nearly 74% of the markers (23,490 of 31,739 SNPs) in the first dataset were polymorphic across the 117 template control DNA samples. Genotyping errors across the multiple DNA samples of each of the 15 accessions varied from 0.2 to 3.1%, with an overall average of 0.8%. Genotyping error between pairs of DNA samples of the same accession exceeded 1% only in three (WAB0002367, WAB0013445, and WAB0000026) of the 15 accessions (Fig. 1). In the second dataset, 87% of the markers (27,645 of 31,739 SNPs) were polymorphic across the 3134 accessions, each SNP with a minor allele frequency ranging from 0.01 to 0.499 (data not shown). Ninety-seven of the 3134 accessions were misclassified (Supplementary Table S1), which accounts for 3.1% of the total germplasm evaluated in this study. The 97 misclassified accessions included 64 *O. glaberrima*, 22 *O. sativa*, 7 *O. barthii*, and 4 *O. longistaminata*. As shown in Supplementary Fig. S1 and Figs. 2 and 3, the misclassified *O. barthii* accessions clustered together with *O. longistaminata* (1), *O. glaberrima* (2), and *O. sativa* (4), whereas those misclassified accessions from *O. longistaminata* were similar either to *O. glaberrima* (1) or *O. barthii* (3). The 64 misclassified *O. glaberrima* accessions were clustered with the lowland *O. sativa* (44) and upland *O. sativa* (22) accessions (Fig. 2), while all 22 misclassified *O. sativa* accessions were clustered together with *O. glaberrima* (Fig. 3). Of the 97 misclassified samples, 37 samples were regenerated seed lots, which included *O. glaberrima* (32), *O. longistaminata* (1), and *O. sativa* (4).

### Diagnostic marker identification

To develop a smaller set of species- or group-specific markers for low-cost and routine QC genotyping, we searched for diagnostic SNPs across 3037 of the 3134 accessions after excluding the 97 misclassified samples. Supplementary Table S2 summarizes the 35 pairs of comparisons that involved the four species, three groups of *O. sativa* (*O. sativa* spp. *indica* and *japonica* and interspecifics) and two ecologies (lowland and upland). The number of diagnostic SNPs identified between pairs of species or groups varied from none to 5640. We first searched for diagnostic SNPs between indigenous African species complex (*O. glaberrima*, *O. barthii*, and *O. longistaminata*; *N* = 2418) as one group and the Asian rice (*O. sativa*, *N* = 619) as the second group and identified 156 diagnostic SNPs (Supplementary Table S3) that clearly revealed contrasting haplotypes between the two groups. One hundred and thirty-six of the 156 diagnostic SNPs were mapped to the 12 rice chromosomes, while the remaining 20 SNPs were not assigned to any chromosome. The number of mapped diagnostic SNPs between the African species complex and the Asian rice varied from 1 on chromosome 8 to 29 on chromosome 2. In the second step, we searched for diagnostic SNPs that discriminated between pairs of the three African species complex and between *O. sativa* groups (*O. sativa* spp. *indica* and *japonica* or lowland and upland ecologies). We found that all *O. longistaminata* accessions differed from *O. barthii* and *O. glaberrima* at 649 and 141 SNPs, respectively, of which 131 SNPs were common in both comparisons (Supplementary Table S2, Supplementary Table S2). No diagnostic SNP was identified between *O. barthii* and *O. glaberrima*. Since no marker was found to be diagnostic between *O. glaberrima* and *O. barthii*, the 131 SNPs should be sufficient to serve as diagnostic markers between *O. longistaminata* and *O. glaberrima*/*O. barthii* accessions. One hundred and ten of the 131 were mapped across the 12 rice chromosomes, with each chromosome consisting of 4 to 29 diagnostic SNPs. Sets of 30 and 45 SNPs discriminating *O. sativa* accessions to lowland or upland ecologies and *O. sativa* spp. *indica* or *japonica*, respectively, were identified (Supplementary Table S2, Supplementary Table S3). The 30 diagnostic SNPs were common across the two ecologies and the two ecotypes, whereas 15 SNPs were diagnostic only between *O. sativa* spp. *indica* and *japonica*. No diagnostic SNP was found between NERICA and *O. sativa* spp. *indica* and *japonica*, which is expected due to the high genetic similarity of NERICAs with their recurrent *O. sativa* parents with a clear population structure corresponding either to the lowland or upland ecology (Supplementary Fig. S1 and Fig. 2).

**Fig. 4:**
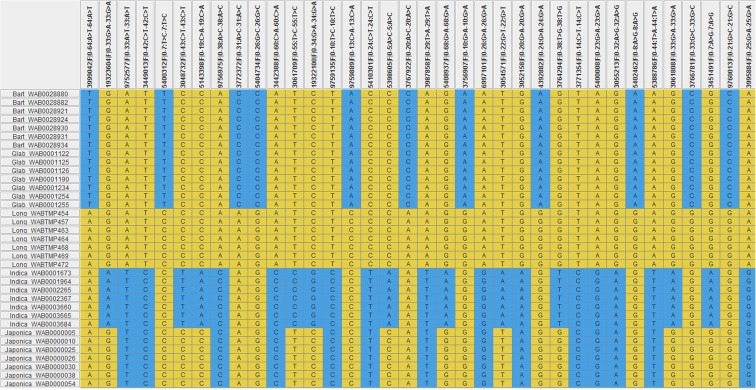
Haplotype pattern of 36 diagnostic SNP markers recommended for quality control genotyping between the three African species complex and *O. sativa* (14 SNPs), between *O. longistaminata* and *O. barthii*/*O. glaberrima* (11 SNPs) and between lowland *O. sativa* spp. *indica* and upland *O. sativa* spp. *japonica* (11 SNPs). Each species or ecotype is represented by seven randomly selected accessions, which are listed in rows on the left side with prefix (Glab *O. glaberrima*, Bart *O. barthii*, Long *O. longistaminata*, Indica *O. sativa* spp. *indica*, japonica *O. sativa* spp. *japonica*), followed by accession number. The SNP IDs are listed as column headings. Each column has two alleles that are shaded either in orange or blue. See Supplementary Table S1 for details on accessions, Supplementary Table S3 for SNP summary, and Supplementary Table S4 for the genotype data of all diagnostic markers across all 3037 accessions (excluding misclassified accessions)

Overall, the total number of diagnostic markers identified across the three sets of germplasm was 332 SNPs, each with MAF and polymorphism information content (PIC) varying from 0.005 to 0.223 and from 0.004 to 0.487, respectively. Supplementary Fig. 2 shows the chromosomal distribution of 285 of the 332 diagnostic SNPs that were mapped across the 12 rice chromosomes. Based on chromosomal positions and a minimum MAF of 0.175 and a PIC value of 0.150, we recommend 36 of the 285 diagnostic SNPs mapped across the 12 rice chromosomes for low-cost quality control genotyping (Table 1, Supplementary Table S3). The genotype data of the 36 diagnostic SNPs across the 3037 accessions (excluding the 97 outliers) are given in Supplementary Table S4. As shown in Fig. 4 and Supplementary Table S3, we selected a subset of 14, 11, and 11 SNPs for unambiguous haplotype discrimination of the three African species complex from Asian rice, *O. longistaminata* from both *O. barthii*/*O. glaberrima*, and lowland *O. sativa* spp. *indica* from upland *O. sativa* spp. *japonica*, respectively. To minimize genotyping cost per sample for uniplex assays, however, a smaller subset of even eight diagnostic SNPs per group of germplasm are sufficient for routine QC genotyping, which is discussed in detail in the next section.

**Table 1:** Summary of the 36 diagnostic SNPs recommended for routine quality control genotyping in three sets of rice germplasm. (See Supplementary Table S3 for details, including major and minor alleles and sequences)

Clone (SNP) ID	Chromosome*	Physical position (bp)*	Minor allele frequency	Polymorphism information content (PIC)	Comment**
19323604|F|0-33:G>A-33:G>A	1	13,006,094	0.175	0.179	Diagnostic between lowland *O. sativa* spp. *indica* and upland *japonica*
3048732|F|0-43:C>T-43:C>T	2	33,878,778	0.175	0.155	Diagnostic between lowland *O. sativa* spp. *indica* and upland *japonica*
5143398|F|0-19:C>A-19:C>A	3	878,615	0.176	0.248	Diagnostic between lowland *O. sativa* spp. *indica* and upland *japonica*
3061709|F|0-55:T>C-55:T>C	4	27,782,473	0.175	0.150	Diagnostic between lowland *O. sativa* spp. *indica* and upland *japonica*
19322100|F|0-34:G>A-34:G>A	5	7,309,276	0.175	0.192	Diagnostic between lowland *O. sativa* spp. *indica* and upland *japonica*
5398605|F|0-5:A>C-5:A>C	6	18,012,850	0.176	0.177	Diagnostic between lowland *O. sativa* spp. *indica* and upland *japonica*
5408937|F|0-68:G>A-68:G>A	7	7,357,816	0.175	0.181	Diagnostic between lowland *O. sativa* spp. *indica* and upland *japonica*
3054571|F|0-22:G>T-22:G>T	8	18,620,171	0.177	0.168	Diagnostic between lowland *O. sativa* spp. *indica* and upland *japonica*
3764294|F|0-38:T>G-38:T>G	9	18,312,602	0.176	0.159	Diagnostic between lowland *O. sativa* spp. *indica* and upland *japonica*
3061808|F|0-33:G>A-33:G>A	11	11,776,508	0.176	0.257	Diagnostic between lowland *O. sativa* spp. *indica* and upland *japonica*
3451491|F|0-7:A>G-7:A>G	12	12,303,324	0.176	0.161	Diagnostic between lowland *O. sativa* spp. *indica* and upland *japonica*
3999042|F|0-64:A>T-64:A>T	1	3,338,923	0.225	0.343	Diagnostic between *O. longistaminata* and *O. glaberrima*/*O. barthii*
5400312|F|0-7:T>C-7:T>C	2	14,337,444	0.224	0.346	Diagnostic between *O*. *longistaminata* and O*. glaberrima*/*O. barthii*
3772372|F|0-31:A>C-31:A>C	3	32,703,627	0.222	0.341	Diagnostic between *O. longistaminata* and *O. glaberrima*/*O. barthii*
5404734|F|0-26:G>C-26:G>C	4	12,064,222	0.224	0.341	Diagnostic between *O. longistaminata* and *O. glaberrima/O. barthii*
9759889|F|0-13:C>A-13:C>A	5	23,874,724	0.224	0.340	Diagnostic between *O. longistaminata* and *O. glaberrima/O. barthii*
3767922|F|0-20:A>C-20:A>C	6	29,415,564	0.224	0.342	Diagnostic between *O. longistaminata* and *O. glaberrima/O. barthii*
3756907|F|0-10:G>A-10:G>A	7	22,969,163	0.223	0.252	Diagnostic between *O. longistaminata* and *O. glaberrima/O. barthii*
4392882|F|0-24:G>A-24:G>A	9	17,267,685	0.225	0.470	Diagnostic between *O. longistaminata* and *O. glaberrima/O. barthii*
5402462|F|0-8:A>G-8:A>G	10	21,079,827	0.224	0.339	Diagnostic between *O. longistaminata* and *O. glaberrima/O. barthii*
3766781|F|0-33:G>C-33:G>C	11	19,638,886	0.223	0.342	Diagnostic between *O. longistaminata* and *O. glaberrima/O. barthii*
9760013|F|0-21:G>C-21:G>C	12	17,069,058	0.225	0.487	Diagnostic between *O. longistaminata* and *O. glaberrima/O. barthii*
9752577|F|0-33:A>T-33:A>T	1	40,671,128	0.219	0.344	Diagnostic SNP between Africa rice species complex and Asian rice
3449013|F|0-42:C>T-42:C>T	2	2,687,374	0.219	0.382	Diagnostic SNP between Africa rice species complex and Asian rice
9756975|F|0-38:A>C-38:A>C	3	21,681,288	0.219	0.305	Diagnostic SNP between Africa rice species complex and Asian rice
3442388|F|0-60:C>A-60:C>A	4	23,879,007	0.219	0.262	Diagnostic SNP between Africa rice species complex and Asian rice
9759135|F|0-10:T>C-10:T>C	5	20,316,123	0.219	0.318	Diagnostic SNP between Africa rice species complex and Asian rice
5410301|F|0-24:C>T-24:C>T	6	5,959,340	0.219	0.251	Diagnostic SNP between Africa rice species complex and Asian rice
3987858|F|0-29:T>A-29:T>A	7	4,661,018	0.219	0.349	Diagnostic SNP between Africa rice species complex and Asian rice
6997101|F|0-26:G>A-26:G>A	8	8,968,236	0.219	0.384	Diagnostic SNP between Africa rice species complex and Asian rice
3052158|F|0-20:G>A-20:G>A	9	1,894,850	0.219	0.252	Diagnostic SNP between Africa rice species complex and Asian rice
3771354|F|0-14:C>T-14:C>T	9	18,345,726	0.219	0.339	Diagnostic SNP between Africa rice species complex and Asian rice
5400008|F|0-23:G>A-23:G>A	9	21,792,333	0.219	0.357	Diagnostic SNP between Africa rice species complex and Asian rice
3055213|F|0-32:A>G-32:A>G	10	10,645,458	0.219	0.324	Diagnostic SNP between Africa rice species complex and Asian rice
5388786|F|0-44:T>A-44:T>A	11	2,335,078	0.219	0.386	Diagnostic SNP between Africa rice species complex and Asian rice
3995884|F|0-25:G>A-25:G>A	12	25,433,207	0.219	0.332	Diagnostic SNP between Africa rice species complex and Asian rice

*Chromosome numbers and physical positions in base pairs (bp) are based on Rice_v9

**African rice species complex refers to *O. glaberrima*, *O. barthii*, and *O. longistaminata*

## Discussion

Gene bank managers are challenged to ensure the accurate identification of species and maintaining the genetic integrity of collections by preventing human errors during routine gene bank operations, including labeling errors and admixtures during seed handling. Many gene banks do not have the resources to assess every collection for genetic diversity, correct origins, and species classification (Mason et al. 2015). Using a set of 93 to 235 markers, previous studies conducted on rice germplasm conserved at AfricaRice gene bank have reported the presence of admixture between *O. glaberrima* and *O. sativa* (Semon et al. 2005) and between *O. glaberrima* and *O. barthii* (Orjuela et al. 2014). However, none of those studies have provided evidence of possible human error on such types of admixture. To the best of our knowledge, this is the first extensive study that has explored the extent of human error using large set of rice germplasm and identified species- and ecotype-specific diagnostic SNPs for low-cost and high-throughput QC genotyping. Overall, we found that 3.1% of the 3134 accessions used in the present study were misclassified (Supplementary Table S1). Such misclassification could be due to genotyping errors caused by the DArTseq technology, errors during germplasm collection, and/or during routine gene bank operations. The quality of DArTseq markers is assessed based on call rates and reproducibility scores of template control DNA samples, which are provided by DArT Pty Ltd. (the genotyping service provider). The reproducibility score of DArTseq markers is the proportion of technical replicate assay pairs for which the marker score is consistent, which has been reported to be 99–100% (Baloch et al. 2017; Melville et al. 2017). In such cases, genotyping error accounts for a maximum of 1% only. In the current study, the average genotyping error between pairs of template control DNA samples from 15 accessions was 0.8%, which translates into an average reproducibility of 99.2% (range 96.9–99.8%). Between pairs of DNA samples from three accessions, however, larger error rates were noted (Fig. 1). Overall, the average genotyping error for DArTseq SNPs in our study was greater than the values reported in other studies using the same platform (Baloch et al. 2017; Melville et al. 2017) but was lower than the 2.0–2.4% reported using other SNP genotyping platforms (Yan et al. 2010; Semagn et al. 2014) and DNA sequencing (Cubry et al. 2018). Our results together with others suggest a very minimal effect of genotyping error on the proportion of misclassification observed in the present study.

To trace the source of human errors during routine gene bank operations, we compared the genotypic data of the original collections and regenerated seed lot of 37 accessions (Supplementary Table S1). In 35 of the 37 original seed lots, we found that accessions originating from the same species clustered as expected, while two original seed lots were misclassified, probably due to species misclassification during germplasm collection. All the 37 regenerated seed lots were misclassified, which might be caused by labeling errors made during seed regeneration/multiplication and seed processing/handling. The proportion of misclassification observed in our study was much lower than the 5–21% misclassification reported in other studies (Buso et al. 2001; Girma et al. 2012; Orjuela et al. 2014; Mason et al. 2015). Orjuela et al. (2014) reported misclassification of 13 of 266 *O. glaberrima* accessions (4.9%), which were supposed to be *O. sativa*; however, the proportion of misclassification in their study seems over 20%, as there were several *O. barthii* accessions that were clustered with *O. glaberrima* in both neighbor-joining phylogenetic analysis and PCA. Buso et al. (2001) studied 230 cultivated and wild *Oryza* species using random amplified polymorphic DNA, flow cytometry, and chromosome counting and reported 8% of misclassification either taxonomically or as a result of contamination. Mason et al. (2015) genotyped 180 lines from five *Brassica* species sourced from the Australian Grains Genebank using the Illumina Infinium Brassica 60K SNP array, which included 76 suspected misclassified samples and 104 randomly selected lines from the germplasm collection. The authors found out that (i) 18% of the 180 lines were misclassified based on species; (ii) 30% of the 76 suspected samples were misclassified; (iii) 9% of the randomly selected samples were misclassified; (iv) several individuals were found to be the product of interspecific hybridization events; and (vi) SNP markers proved to be effective at confirming species identity. Using 53 phenotypic descriptors, approximately 21% of 3156 yam accessions were found misidentified (not true-to-type individuals) (Girma et al. 2012). Such types of errors can easily be corrected using diagnostic markers and by implementing routine QC genotyping.

To date, a systematic genotyping QC method has been developed and implemented only in maize (Semagn et al. 2012; Ertiro et al. 2015; Chen et al. 2016) to minimize errors associated with genetic purity and genetic identity/authentication. In maize, the focus on QC genotyping was to maintain high genetic purity and consistent genetic identity/authenticity of popular parental lines to minimize use of incorrect parents in breeding and genetic studies. In study by the International Maize and Wheat Improvement Center (CIMMYT), for example, researchers compared results from QC genotyping using a smaller set of preselected SNPs with field notes for 280 seed sources of 40 popular parental lines (inbred and doubled haploid lines) and discarded those sources that showed deviation from expectation in terms of purity and identity (Ertiro et al. 2015). In all cases, the decisions made using the preselected SNP markers were as accurate as the field notes recorded on various phenotypic traits. The QC genotyping based on markers was cheaper and faster than evaluating various seed lots in field plots over a period of 3–4 months. In the present study, we used a similar approach as that of maize and verified the 37 accessions genotyped from the original and regenerated seed lots by growing the two seeds lots side by side at the AfricaRice experimental field in Cotonou, Benin (Ndjiondjop et al. 2017). By comparing phenotypic traits recorded on each seed lot with the SNP data, we have corrected the misclassified original seed lots and recommended discarding the regenerated seed lots.

SNP genotype data can be obtained using one of the numerous uniplex, multiplex, and genotyping by sequencing methods (Semagn et al. 2015). For QC genotyping, a smaller subset of SNPs need to be selected for low-cost uniplex SNP quality control analysis based on ease of scoring with unambiguous separation of homozygous and heterozygous genotypes, minor allele frequency, polymorphism information content, and uniform distribution across chromosomes (Semagn et al. 2012). Using these selection criteria, we recommend between two to three SNPs per chromosome to serve as diagnostic markers in each of the three grups of germplasm. These three groups of germplasm refer to those diagnostic SNPs that separated the three African species complex from the Asian rice, those separating *O. longistaminata* from *O. glaberrima/O. barthii*, and those discriminating lowland *O. sativa* spp. *indica* from upland *O. sativa* spp. *japonica*. Of the list of diagnostic markers summarized in Supplementary Table S3 and Supplementary Fig. S2, we recommend developing Kompetitive Allele-Specific PCR (KASP) assays (Semagn et al. 2014) for 36 diagnostic SNPs, with 11–14 SNPs per group of germplasm (Table 1). Researchers from CGIAR and national agricultural research systems (NARS) in developing countries have a contractual agreement with KASP genotyping service providers that costs about US$ 1.5–2.5 and US$ 4–5 per sample for a set of 10 and 24 SNPs, respectively, which includes both DNA extraction and SNP genotyping (http://excellenceinbreeding.org/module3). The actual cost per sample varies with the total number of samples to be genotyped. To utilize such services, users can choose 24 of the 36 SNPs (Fig. 4 and Supplementary Table S3) that we recommended for routine QC genotyping in rice. In cases where the QC genotyping should be done within *O. sativa* germplasm, only 8–11 SNPs that discriminate the lowland *O. sativa* spp. *indica* from the upland *O. sativa* spp. *japonica* should be used (Supplementary Table S3). The same is true if the purpose is to discriminate the African species complex from Asian rice or the perennial *O. longistaminata* from the annual *O. barthii* and *O. glaberrima*. Our inability to identify any diagnostic marker between *O. barthii* and *O. glaberrima* was not unexpected. *O. glaberrima* is thought to have evolved from its wild ancestor *O. barthii* through selection (Ogbe and Williams 1978; Linares 2002) and there is evidence showing a wide range of intermediate types between them (Ogbe and Williams 1978). Some diagnostic phenotypic characters such as growth habit, spikelets shattering, and hairiness both on awn and spikelets have been identified. However, such phenotypic traits are often affected by genotype-by-environment interaction and are not conclusive across diverse accessions. We also did not find any diagnostic SNPs between NERICA and *O. sativa* spp. *indica* and between NERICA and *O. sativa* spp. *japonica*. NERICAs were derived from interspecific crosses between *O. sativa* and *O. glaberrima* and selected for adaptation to upland, the rainfed lowland, and the irrigated lowland ecologies across West and Central Africa (Maclean et al. 2002). NERICAs are predominantly *O. sativa* (*indica* or *japonica*) background, which is evident from the low proportion of *O. glaberrima* genome estimated using microsatellite markers (Ndjiondjop et al. 2008) and the moderate genetic differentiation observed between lowland NERICA and lowland *O. sativa* spp. *indica* (11.5%) and between upland NERICA and upland *O. sativa* spp. *japonica* (6.6%) using SNP markers (Ndjiondjop et al. 2018). Results from this study would be highly relevant for rice breeders, gene bank managers, and seed system specialists.

## Conclusions

Our results demonstrate the usefulness of genomic tools not only to evaluate the genetic variation and population structure of germplasm conserved in gene banks, but also to detect and correct human errors that could occur at some stage during germplasm collections and routine gene bank operations. Uniplex or multiplex SNP assays can be developed from the sequence information of either all diagnostic SNPs or a subset of preselected species and subspecies (ecotype) diagnostic SNPs that we recommended for routine QC genotyping of at least rice germplasm that are high in demand for distribution to users. About 24 diagnostic SNPs should be sufficient for routine QC genotyping, which includes a set of 8 diagnostic SNPs to discriminate the three African species complex from the *O. sativa*, a second set of 8 SNPs to separate *O. longistaminata* from *O. glaberrima/O. barthii*, and a third set of 8 SNPs to discriminate lowland *O. sativa* spp. *indica* from upland *O. sativa* spp. *japonica*. To the best of our knowledge, this is the first extensive study that identified species- and ecotype-specific diagnostic SNPs across multiple *Oryza* species for low-cost and high-throughput QC genotyping. Using such diagnostic SNP markers, gene bank researchers can determine the identity of their germplasm collections and track misidentification, mislabeling, physical contamination, and loss of genetic integrity.

## Electronic supplementary material

Supplementary Table S1 (XLSX 188 kb)

Supplementary Table S2 (DOCX 14 kb)

Supplementary Table S3 (XLSX 74 kb)

Supplementary Table S4 (XLSX 355 kb)

Supplementary Fig. S1 (DOCX 59 kb)

Supplementary Fig. 2 (DOCX 73 kb)
