# Supplementary material for: Development of species diagnostic SNP markers for quality control genotyping in four rice (Oryza L.) species
Source: Mol Breed. 2018 Oct 24;38(11):131. doi: 10.1007/s11032-018-0885-z (PMC6208651; doi:10.1007/s11032-018-0885-z)
Supplement: Supplementary file 2 — (DOCX 14 kb) [file 11032_2018_885_MOESM2_ESM.docx]

**Supplementary Table S2.** Summary of the number of diagnostic marker identified between pairs of species/groups based on 3,037 accessions genotyped with 31,739 SNPs.

| Group | *O. barthii* (N = 44) | *O. longistaminata* (N = 16) | *O. glaberrima* (N= 2,358) | *O. barthii*, *O. longistaminata* and *O. glaberrima* (N = 2,418) | *O. sativa* spp. indica (N= 442) | *O. sativa* spp. japonica (= 94) | Upland *O. sativa* and upland NERICA (N = 114) |
| --- | --- | --- | --- | --- | --- | --- | --- |
| *O. barthii* (N = 44) |  |  |  |  |  |  |  |
| *O. longistaminata* (N = 16) | 649 |  |  |  |  |  |  |
| *O. glaberrima* (N= 2358) | 0 | 141 |  |  |  |  |  |
| *O. glaberrima* and *O. longistaminata* (N= 2,374) | 0 | NA | NA |  |  |  |  |
| *O. glaberrima and O. barthii* (N= 2,402) | NA | 131 | NA |  |  |  |  |
| *O. sativa* spp. indica (N= 442) | 2,149 | 1,666 | 641 | 449 |  |  |  |
| *O. sativa* spp. japonica (= 94) | 5,640 | 4,458 | 1,394 | 1,015 | 45 |  |  |
| NERICA (N = 83) | 3,925 | 2,974 | 771 | 507 | 0 | 0 | NA |
| Lowland *O. sativa* and lowland NERICA (N = 505) | 1,585 | 1,226 | 493 | 347 | NA | 31 | 30 |
| Upland *O. sativa* and upland NERICA (N = 114) | 4,461 | 3,555 | 1,106 | 811 | 41 | NA | NA |
| All *O. sativa* and NERICA (N = 619) | 862 | 655 | 230 | 156 | NA | NA | NA |
