# Supplementary material for: Development of species diagnostic SNP markers for quality control genotyping in four rice (Oryza L.) species
Source: Mol Breed. 2018 Oct 24;38(11):131. doi: 10.1007/s11032-018-0885-z (PMC6208651; doi:10.1007/s11032-018-0885-z)
Supplement: Supplementary file 5 — (DOCX 59 kb) [file 11032_2018_885_MOESM5_ESM.docx]

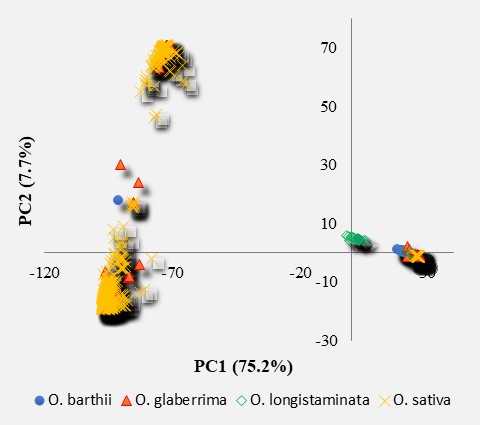


Lowland *O. sativa*

Upland *O. sativa*

**Supplementary Fig. S1** Plots of PC1 and PC2 from principal component analysis of 3,134 accessions representing *O. barthii* (51), *O. glaberrima* (2,422), *O. longistaminata* (20), and *O. sativa* and NERICA (641 accessions) using 27,645 polymorphic SNPs. Note markedly lower genetic diversity within and between the three African species complex (*O. barthii,* *O. longistaminata* and *O. glaberrima*) on the right side of the quadrant as compared to the Asian rice (*O. sativa*). Lowland and upland genotypes are primarily O. sativa spp. indica and japonica, respectively.
