# Supplementary material for: Development of species diagnostic SNP markers for quality control genotyping in four rice (Oryza L.) species
Source: Mol Breed. 2018 Oct 24;38(11):131. doi: 10.1007/s11032-018-0885-z (PMC6208651; doi:10.1007/s11032-018-0885-z)
Supplement: Supplementary file 6 — (DOCX 73 kb) [file 11032_2018_885_MOESM6_ESM.docx]

**Supplementary Fig.** 2 Physical position of 285 of the 332 diagnostic SNPs identified for three groups: (a) red font: diagnostic SNPs between the three African species complex (*O. glaberrima*, *O. barthii* and *O. longistaminata*) and Asian rice (*O. sativa*); (b) black font: diagnostic SNPs between *O. longistaminata* and *O. barthii*/*O. glaberrima*; and (c) green fonts: diagnostic SNPs between *O. sativa* spp. indica and japonica. The 36 diagnostic SNPs recommended for routine quality control genotyping are underlined, with 11-14 diagnostic SNPs per group of germplasm. The vertical position is physical map distance ×10^5^ bp. See Supplementary Table S3 for details on each SNP.
